# Supplementary material for: Unravelling the effect of charge dynamics at the plasmonic metal/semiconductor interface for CO2 photoreduction
Source: Nat Commun. 2018 Nov 26;9:4986. doi: 10.1038/s41467-018-07397-2 (PMC6255847; doi:10.1038/s41467-018-07397-2)
Supplement: Supplementary file 1 — Supplementary Information [file 41467_2018_7397_MOESM1_ESM.pdf]

Supplementary Information

Unravelling the effect of charge dynamics at the plasmonic metal/semiconductor interface for CO_2 photoreduction

L. Collado, A. Reynal, F Fresno, M. Barawi, C. Escudero, V. Perez-Dieste, J. M. Coronado, D. P. Serrano, J R. Durrant, V A. de la Peña O'Shea*

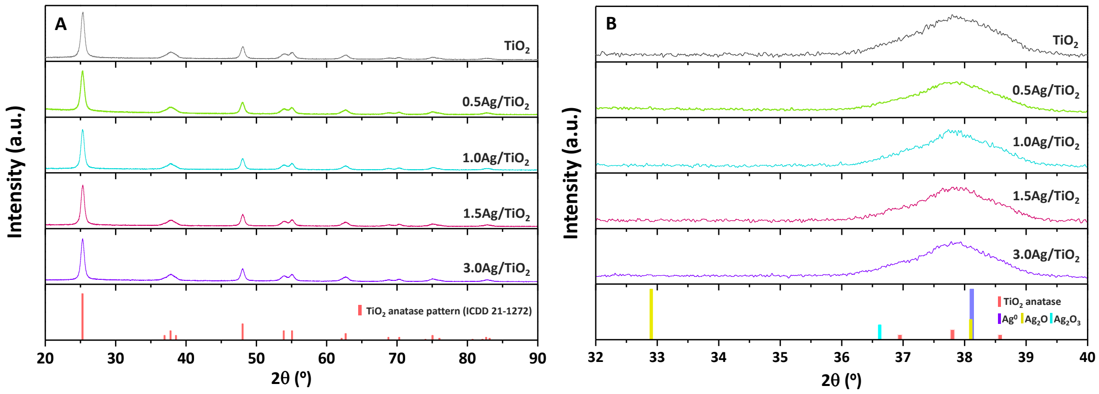

Supplementary Figure 1. XRD profiles of commercial TiO_2 and powdered Ag/TiO_2 (0.5 - 3.0 wt.% Ag) catalysts **(A)**, and enlargement of the Ag/TiO_2 diffraction profiles in the range 2θ = 32 – 40° **(B)**. Diffraction patterns of pure anatase and silver species (Ag^0, Ag_2O and Ag_2O_3) are also included for comparison. XRD studies confirm the only presence of the anatase phase (ICDD No. 21-1272, I41/amd) in bare TiO_2 (Supplementary Figure 1A). No silver phases are observed in Ag/TiO_2 samples, suggesting the presence of small silver and/or silver oxide nanoparticles (Supplementary Figure 1B).

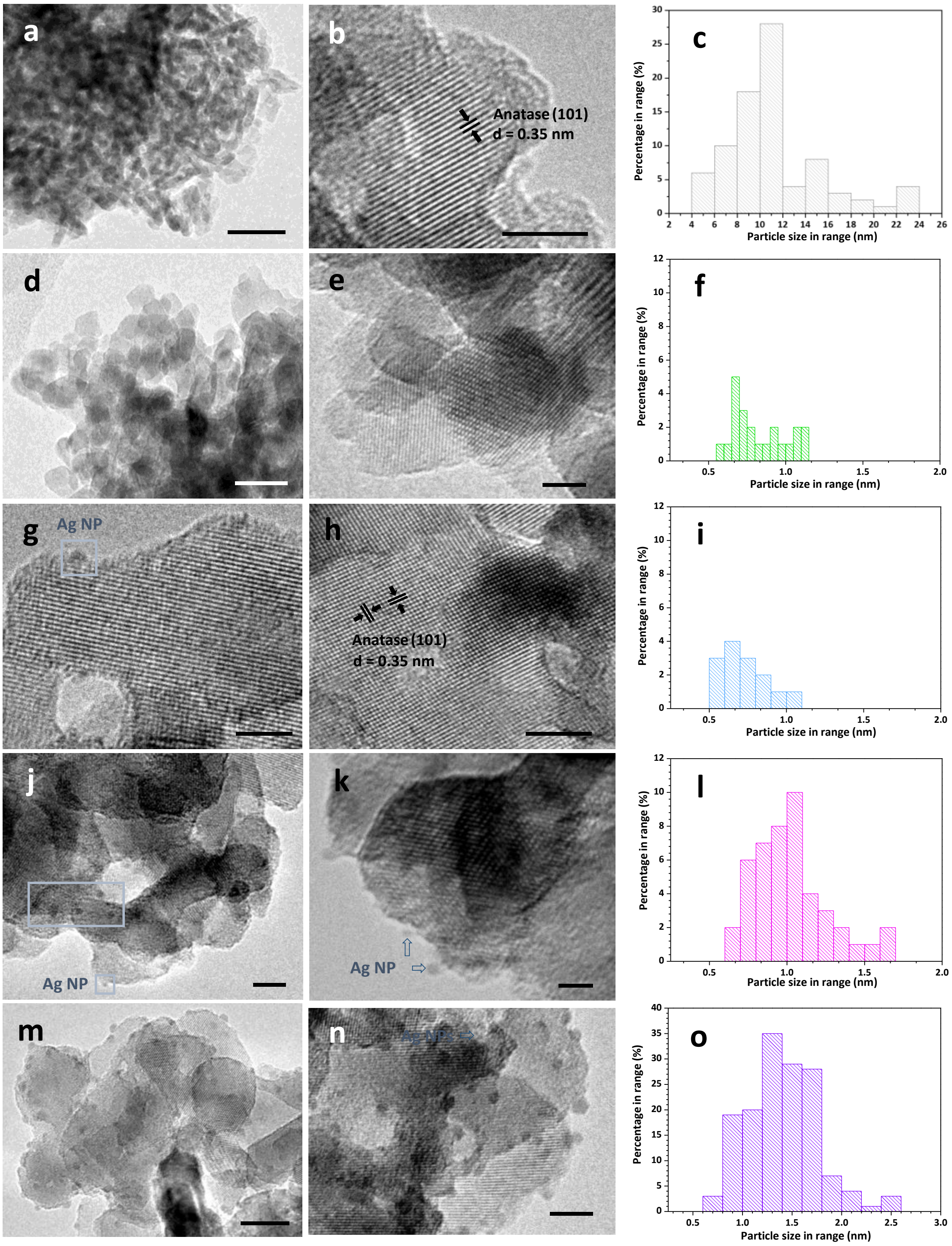

Supplementary Figure 2. TEM micrographs and particle size distribution of commercial TiO_2 **(a-c)** and Ag/TiO_2: 0.5Ag/TiO_2 **(d-f)**, 1.0Ag/TiO_2 **(g-i)**, 1.5Ag/TiO_2 **(j-l)**, 3.0Ag/TiO_2 **(m-o)**. Scale bars: 50 nm (**a**); 25nm (**d**, **j**); 10 nm (**m**); 5 nm (**b**, **e**, **g**, **n**); 2 nm (**h**); 2.5 nm (**k**). TEM analyses confirm the presence of small silver nanoparticles with mean sizes below 1.5 nm, homogeneously distributed over nanosized TiO_2 particles (10–15 nm).

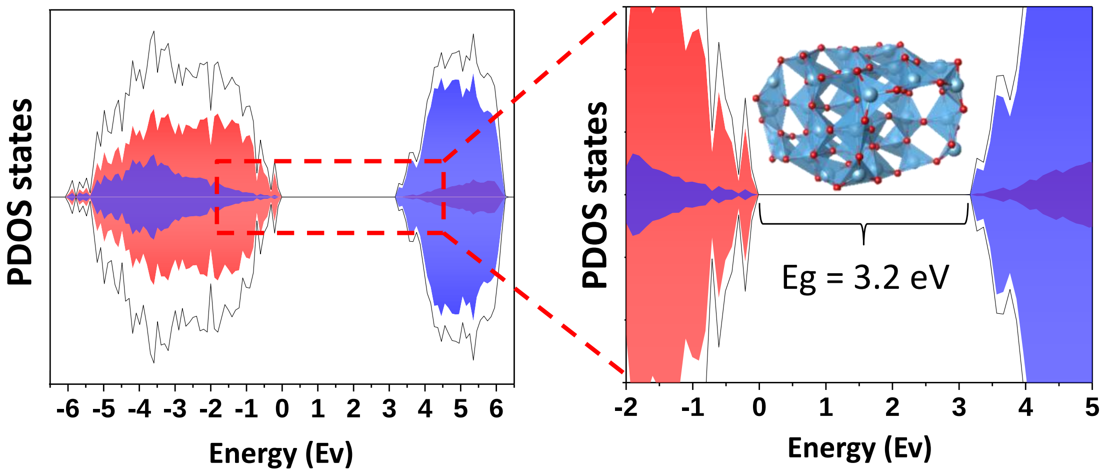

Supplementary Figure 3. Total DOS (black) and atom-projected (PDOS) for a TiO_2 cluster with 38 Ti atoms. Colour: O2p (red) and Ti3d (purple).

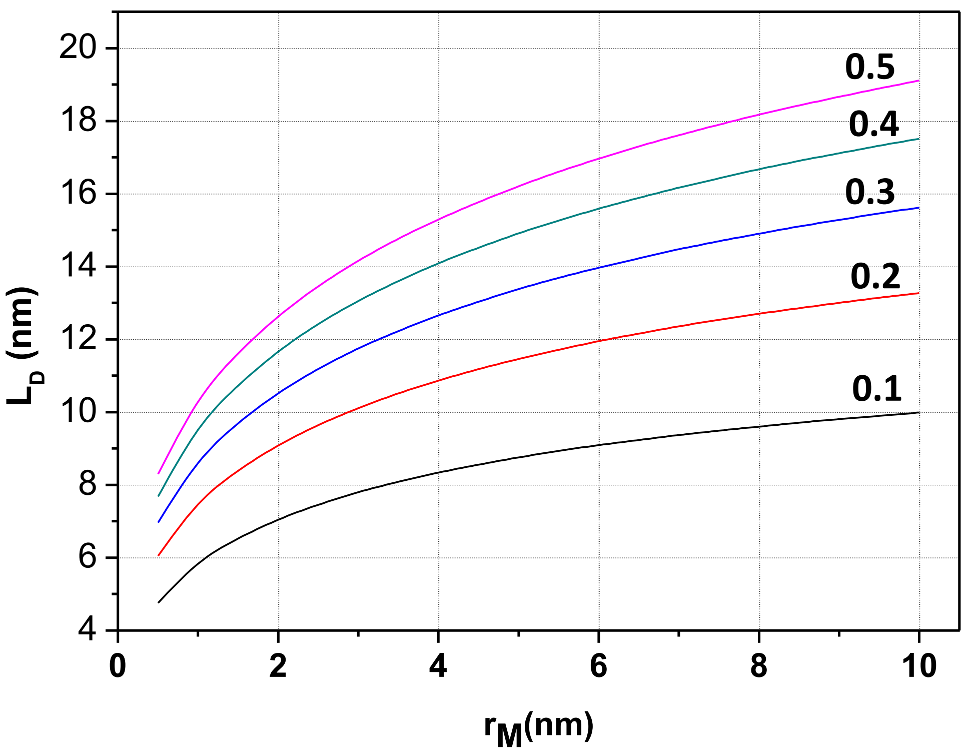

Supplementary Figure 4. Determination of depletion layer thickness (L_D) as function of r_M at different V_{bb}.

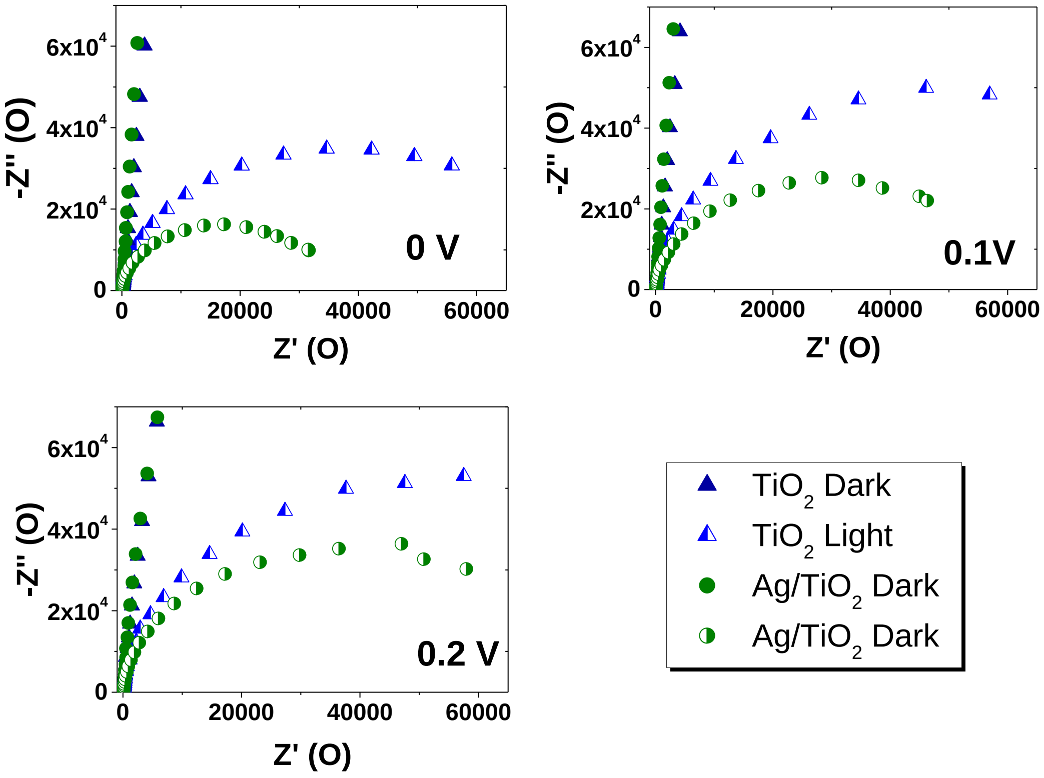

Supplementary Figure 5. Nyquist plots at different bias potential under dark and illumination conditions for both TiO_2 and Ag/TiO_2 samples. Electrochemical Impedance Spectroscopy confirms that Ag/TiO_2 samples exhibit a lower charge transfer resistance and therefore an improved performance under polarization and UV illumination.

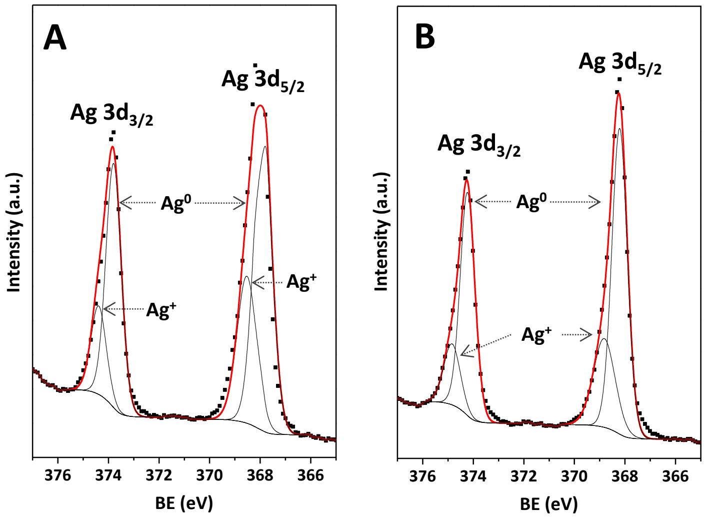

Supplementary Figure 6. Ag3d XPS spectra of 1.5Ag/TiO_2 recorded under UHV in the dark **(A)** and under UV irradiation (365 nm) **(B)**.

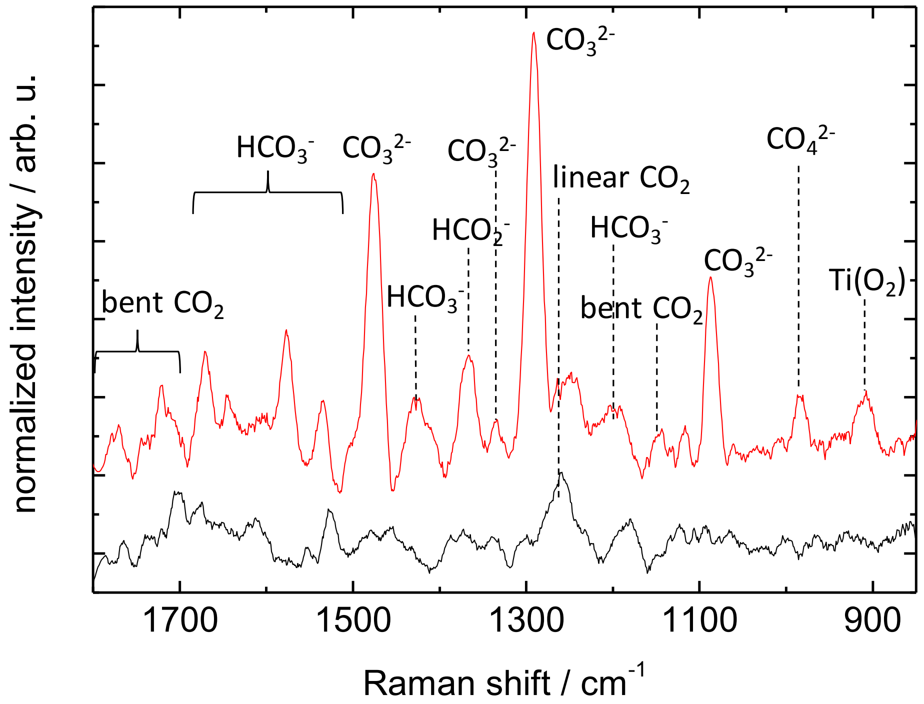

Supplementary Figure 7. Raman spectra of 1.5Ag/TiO$_2$: fresh (black, bottom line) and after CO_2 photoreduction using UV irradiation (red, top).

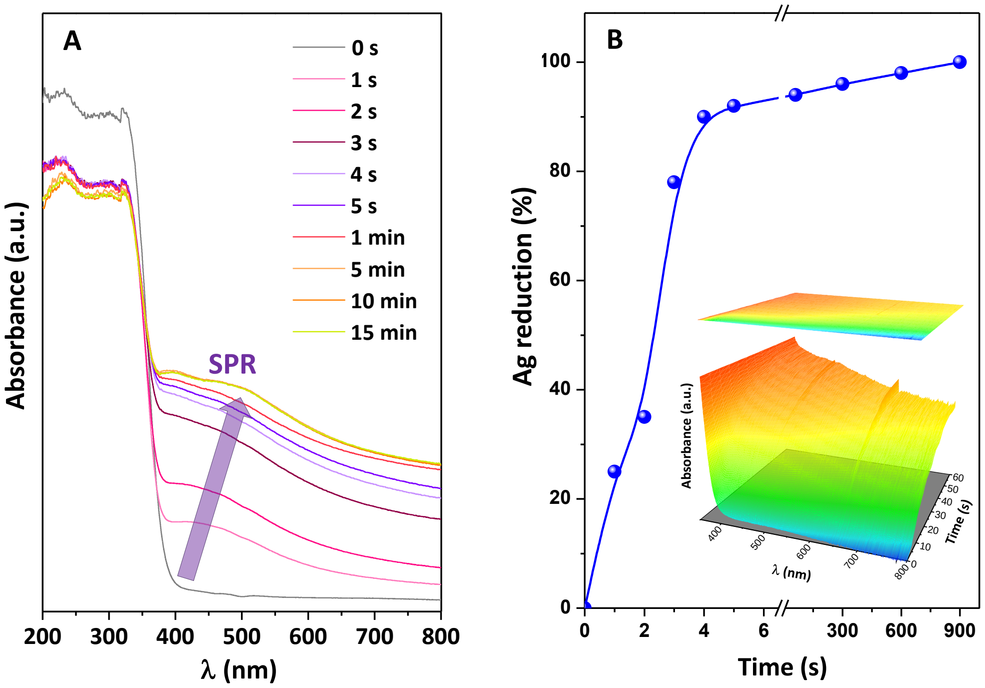

Supplementary Figure 8. Time evolution of the UV-vis spectrum of the 1.5Ag/TiO_2 powdered catalyst over 15 min of UV irradiation (365 nm) **(A)** and degree of silver reduction during this illumination time **(B)**, estimated from the area under the absorption curve at λ > 400 nm.

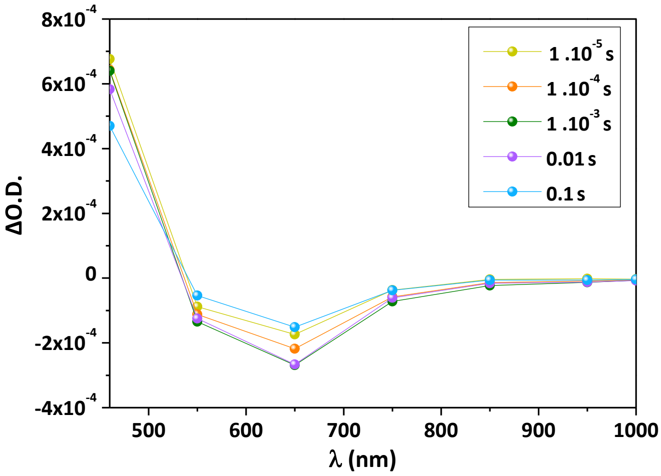

Supplementary Figure 9. Transient absorption spectrum of a Ag/TiO_2 (5 s) film after UV excitation (355 nm, 350 μJ cm^{-2}), measured under N_2 atmosphere.

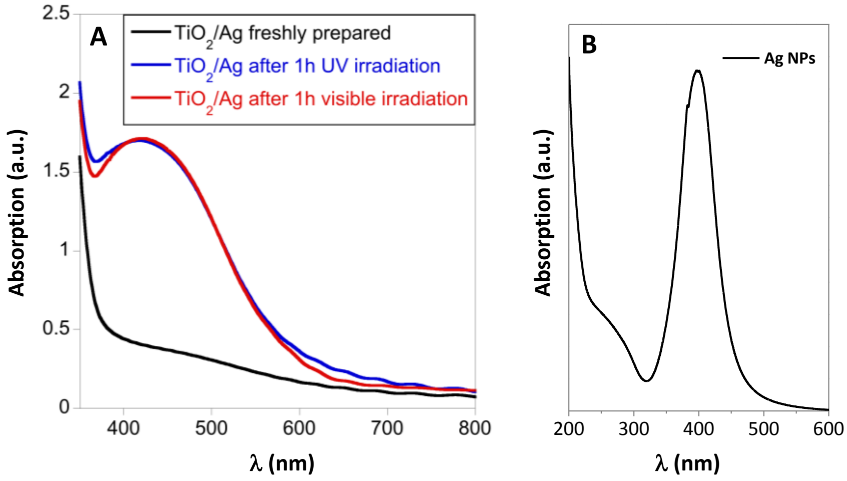

Supplementary Figure 10. **(A)** Absorption spectrum of a 4 μm TiO_2 film loaded with Ag nanoparticles (5 s), using a freshly prepared sample (not irradiated with UV light, black trace), after 1 h of being irradiated with UV light (355 nm, blue trace) and after 1 additional hour of visible light irradiation (532 nm, red trace). The samples were always kept under N_2 atmosphere to avoid degradation. **(B)** Absorption spectrum of an aqueous suspension of Ag nanoparticles (0.02 mg L^{-1}, nanoparticle diameter of 10 nm).

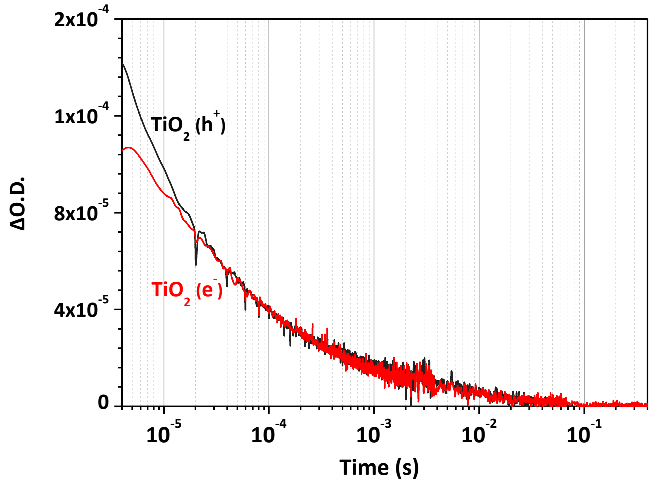

Supplementary Figure 11. Transient absorption decays corresponding to photogenerated electrons (red trace) and holes (black trace) of a 4 μm thick bare TiO_2 film. The decays were acquired under N_2 atmosphere while exciting at 355 nm (350 μJ cm^{-2}, 1 Hz). Electrons were probed at 900 nm and holes at 460 nm.

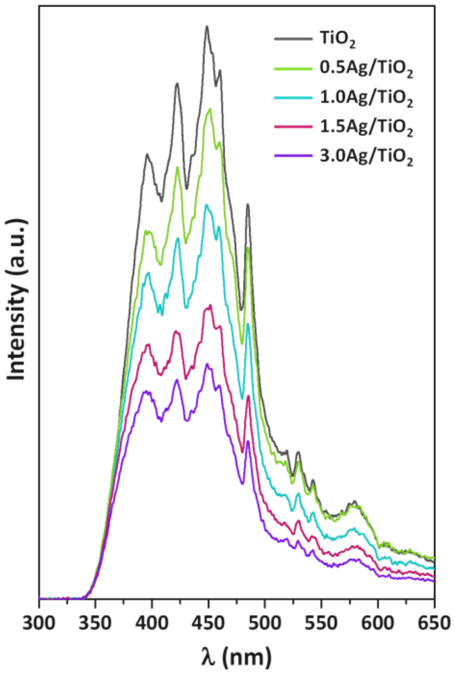

Supplementary Figure 12. Photoluminescence spectra of TiO_2 and Ag/TiO_2 (0.5 - 3.0 wt.%) powdered catalysts (λ_{ex} = 300 nm, cut-off filter at 350 nm).

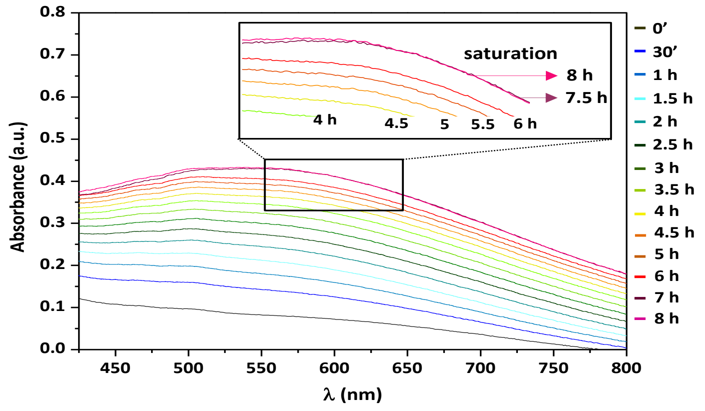

Supplementary Figure 13. Time evolution of the UV-vis DRS spectrum of $1.5Ag/TiO_2$ powdered catalyst under visible illumination at 410 nm.

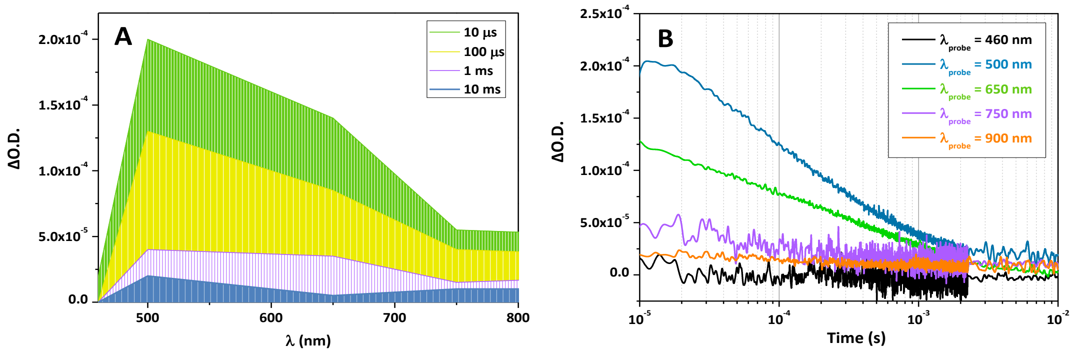

Supplementary Figure 14. Transient absorption spectra **(A)** and decays **(B)** of a Ag/TiO_2 film when exciting with visible light (λ_{ex} = 510 nm) and probing at different wavelengths. The measurements were performed under N_2, using a laser intensity of 350 μJ cm^{-2}.

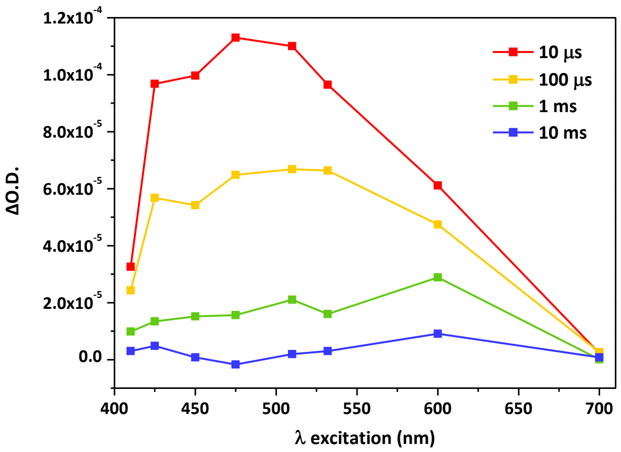

Supplementary Figure 15. Transient absorption spectrum of Ag/TiO_2 while exciting at different wavelengths (400 - 700 nm) and probing at a fixed wavelength of 500 nm. The measurements were performed under N_2, with a laser intensity of 350 µJ cm^{-2} (1 Hz laser pulse frequency). The maximum transient absorption signal of Ag/TiO_2 is observed when exciting at 480 nm, corresponding to the maximum absorption of the Ag SPR.

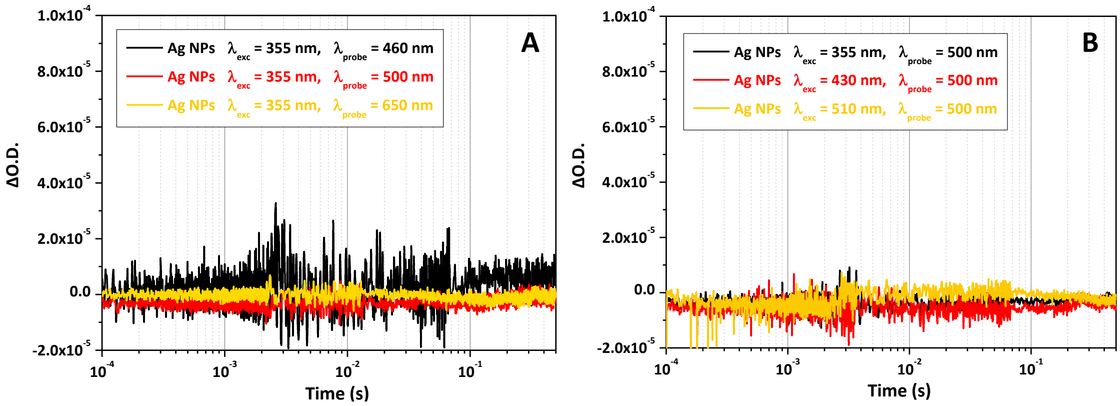

Supplementary Figure 16. Transient absorption data of an aqueous suspension of Ag nanoparticles (0.02 mg L^{-1}, nanoparticle diameter = 10 nm), under N_2. The samples do not show any signal in the range of 460 – 650 nm when excited at 355 nm **(A)** or at longer wavelengths (355, 430 and 510 nm) **(B)**.

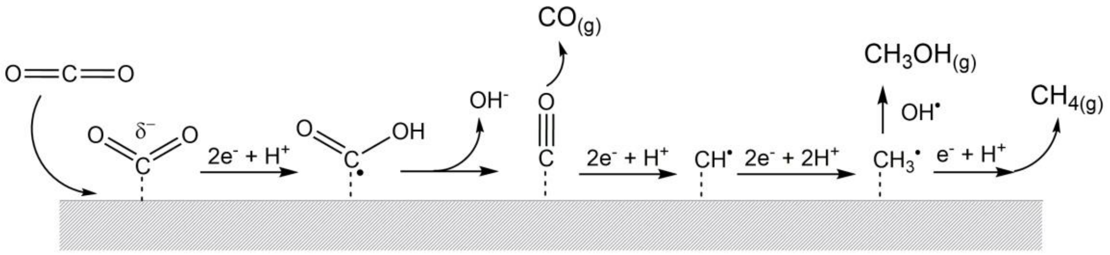

Supplementary Figure 17. Proposed reaction pathways based on the carbene mechanism[16] and on data obtained from *in-situ* experiments.

.

Supplementary Table 1. Physicochemical properties of commercial TiO_2 and powdered Ag/TiO_2 (0.5 - 3.0 wt.% Ag) catalysts.

**Catalyst**	**Ag loading (wt.%)[a]**	**S_{BET} (m^2 g^{-1})[b]**	**V_p (cm^3 g^{-1})[c]**	**D_{Ag} (nm)[d]**
**TiO_2**	-	166	0.44	-
**0.5Ag/TiO_2**	0.43	105	0.40	0.7
**1.0Ag/TiO_2**	0.98	98	0.39	0.8
**1.5Ag/TiO_2**	1.45	90	0.34	1.0
**3.0Ag/TiO_2**	2.92	82	0.33	1.5

[a] Ag content (wt.%) measured by ICP-OES; [b] S_{BET} surface area; [c] Total pore volume at p/p_0 = 0.98; [d] Ag particle size determined by TEM.

Supplementary Table 2. Cumulative productions (μmol g_{cat}^{-1}) over TiO_2 and Ag/TiO_2 (0.5 - 3.0 wt.% Ag) after 15 h of UV irradiation (λ = 365 nm).

Catalyst	H_2	CO	CH_4	CH_3OH
**Commercial TiO_2**	78.9	254.8	5.9	9.8
**0.5Ag/TiO_2**	30.4	6.9	76.4	1.0
**1.0Ag/TiO_2**	31.2	7.1	78.3	1.1
**1.5Ag/TiO_2**	18.6	11.1	86.5	1.4
**3.0Ag/TiO_2**	16.7	6.6	78.6	0.3

Supplementary Table. Literature comparison of the gas-phase CO_2 photoreduction over Ag-loaded catalysts using water as electron donor.

Semiconductor	Operation mode	Light source	T (°C)	CH_4 evolution rate (μmol g^{-1} h^{-1})	CO evolution rate (μmol g^{-1} h^{-1})	Reference
TiO_2	Continuous	UV lamps (4.72 mW cm^{-2})	50	5.8	0.7	This study
MWCNT[a]@TiO_2	Continuous	Visible lamp (15 W)	60	6.3	-	[1]
TiO_2	Continuous	Xe lamp (300 W)	25	1.4		[2]
TiO_2	Batch	Xe lamp (300 W)	-	2.1	1.7	[3]
$AgIO_3$	Batch	Xe lamp (500 W)	25	6.3	6.9	[4]
Ag_2SO_3	Batch	Xe lamp (10.75 mW cm^{-2})	25	3.0	4.3	[5]
TiO_2	Batch	Xe lamp (34.8 mW cm^{-2})	60	0.8	-	[6]
TiO_2	Batch	UV lamp (8 W)	-	2.9	-	[7]
TiO_2	Batch	UV lamp (8 W)	50	0.5	0.7	[8]
$BaZrO_3$	Batch	Xe lamp (300 W)	25	0.3	-	[9]
TiO_2 nanorods	Batch	2 × Xe lamp (300 W)	-	1.1	12.6	[10]
Brookite TiO_2	Batch	Xe lamp (300 W)	-	0.7	14.7	[11]

[a]MWCNT: Multi-walled carbon nanotubes.

Supplementary Table 4. Resistance and capacitance values obtained by the fitting of EIS Nyquist and Bode plots using the electrical circuit present in Figure 4.

E (V)	R, C	TiO_2 Light	TiO_2 Dark	Ag/TiO_2 Light	Ag/TiO_2 Dark
0.0	**R_s (Ω) ±0.5**	23.7	22.3	23.2	23.5
	**R_p (Ω) ±10**	74600	--	36200	--
	**C (μF) ±0.2**	11.4	1.7	11.2	--
0.1	**R_s (Ω) ±0.5**	22.5	21.6	26.1	25.6
	**R_p (Ω) ±10**	108500	--	60500	--
	**C (μF) ±0.2**	10.8	1.2	10.9	1.5
0.2	**R_s (Ω) ±0.5**	22.6	22.5	25.8	25.3
	**R_p (Ω) ±10**	118000	--	77700	--
	**C (μF) ±0.2**	10.7	1.5	10.2	--
0.3	**R_s (Ω)±0.5**	22.4	22.3	25.5	24.3
	**R_p (Ω) ±10**	51600	174000	48000	139000
	**C (μF) ±0.2**	9.8	9.1	9.5	8.8

Supplementary Table 5. In-situ near ambient pressure X-ray photoelectron spectroscopy C1s data collected on the 1.5Ag/TiO_2 sample under the following conditions: UHV; CO_2 and H_2O atmosphere; and CO_2-H_2O atmosphere and UV irradiation.

Component	UHV			CO_2-H_2O			CO_2-H_2O-UV		
	BE (eV)	FWHM	C (%)	BE (eV)	FWHM	C (%)	BE (eV)	FWHM	C (%)
C-H	-	-	-	-	-	-	284.0	1.1	15.8
C-C	284.7	1.1	55.6	284.7	1.2	54.9	284.8	1.1	27.2
C-O	285.5	1.1	35.0	285.8	1.2	31.3	285.8	1.1	17.6
C=O	-	-	-	-	-	-	287.0	1.2	4.8
HCO_3^-	288.6	1.1	6.7	288.9	1.0	8.4	288.9	1.1	13.3
CO_3^{2-}	289.5	1.0	2.7	289.5	1.0	4.9	289.8	1.0	6.8
$CO_2^{\delta-}$	-	-	-	-	-	-	291.9	1.1	3.5
Phys CO_2	-	-	-	-	-	-	292.9	1.0	7.3

BE: Binding energy (eV); FWHM: Full Width at Half Maximum; C: concentration (%).

Supplementary Table 6. NAP-XPS O1s data collected on 1.5Ag/TiO_2 sample under the following conditions: UHV; CO_2 and H_2O atmosphere; and CO_2 - H_2O atmosphere and UV irradiation.

Component	UHV			CO_2-H_2O			CO_2-H_2O-UV		
	BE (eV)	FWMH	C (%)	BE (eV)	FWMH	C (%)	BE (eV)	FWMH	C (%)
O-Ti	529.4	1.0	65.4	529.4	1.0	53.5	529.4	1.0	53.4
O_B	530.1	1.0	20.7	530.1	1.0	22.3	530.1	1.0	22.2
O_H	531.1	1.0	9.8	531.0	1.0	11.7	531.1	1.0	13.5
C-O*	531.7	1.0	3.4	531.8	1.0	6.2	532.0	1.1	6.6
$H_2O_{phys.}$	532.5	1.0	0.7	532.7	1.3	6.4	532.9	1.3	4.3

BE: Binding energy (eV); FWHM: Full Width at Half Maximum; C: concentration (%).

Supplementary Note 1. Electrochemical Characterization

To determine the flat band potential, the capacitance from the space charge layer was calculated from the imaginary part of the impedance. The dependence of C_{SC} on bias potential (V) is described by the Mott-Schottky equation:[12,13]

$$\frac{1}{C_{SC}^2} = \left(\frac{2}{\varepsilon_{SC}\varepsilon_0 N_D e}\right)\left(-[V - V_{FB}] - \frac{k_B T}{e}\right) \qquad \text{Equation (1)}$$

where C_{SC} is the measured differential capacitance per unit area, $\mathcal{E}_{SC}$ = 30 is the dielectric constant for TiO_2 anatase[14], $\mathcal{E}_0$ is the electrical permittivity of vacuum, N_D is the semiconductor carrier density, V is the applied bias potential in volts, k_B is Boltzmann's constant, e the charge of an electron, A the electrode surface (namely, 1 cm^2) and T is temperature (298 K). Therefore, from the $C_{SC}{}^{-2}$ vs. V plot, V_{FB} can be easily obtained by the interception with the x-axis. From the slope of the Mott-Schottky line it is also possible to estimate the mean number of carrier density N_D.

Space charge regions were calculated using the model proposed by Ioannides and Verykios[15] assuming a spherical metal nanoparticle embedded in a semiconductor and based the Schottky approximation were electric field (E(r)) and potential are defined:

$$E(r) = \frac{eN_d}{3\varepsilon_r\varepsilon_0 r^2}\left[(L_D + r_M)^3 - r^3\right] \qquad \text{Equation (2)}$$

$$r_M \leq r \leq D + r_M$$

$$V_{BB}(r) = \frac{eN_d}{\varepsilon_r\varepsilon_0}\left[\frac{(L_D+r_M)^2}{2} - \frac{r^2}{6} - \frac{(L_D+r_M)^3}{3r}\right] \qquad \text{Equation (3)}$$

$$r_M \leq r \leq L_D + r_M$$

Thus, contact potential at the metal/semiconductor interface ($V_{BB}(r_M)$) can be defined as:

$$V_{BB}(r_M) = \frac{eN_d}{\varepsilon_r\varepsilon_0}\left[\frac{(L_D+r_M)^2}{2} - \frac{r^2}{6} - \frac{(L_D+r_M)^3}{3r}\right] \qquad \text{Equation (4)}$$

where e is the electron charge, N_d is the surface density carriers (from equation (1)), $\mathcal{E}_0$ and $\mathcal{E}_{SC}$ = 30 are the dielectric constant for vacuum and TiO_2 anatase,[14] respectively; r_M is the metal radius and L_D is the depletion layer thickness as function of metal particle radius at fixed $V_{BB}(r_M)$. The obtained values in the case of this study are displayed in Supplementary Figure 4.

Supplementary References

1. Gui, M. M., Wong, W. M. P., Chai, S.-P. & Mohamed, A. R. One-pot synthesis of Ag-MWCNT@TiO_2 core–shell nanocomposites for photocatalytic reduction of CO_2 with water under visible light irradiation. *Chem. Eng. J.* **278,** 272–278 (2015).

2. Yu, B., Zhou, Y., Li, P., Tu, W., Li, P., Tang, L., Ye, J. & Zou, Z. Photocatalytic reduction of CO_2 over Ag/TiO_2 nanocomposites prepared with a simple and rapid silver mirror method. *Nanoscale* 11870–11874 (2016).

3. Xie, S., Wang, Y., Zhang, Q., Deng, W. & Wang, Y. MgO- and Pt-promoted TiO_2 as an efficient photocatalyst for the preferential reduction of carbon dioxide in the presence of water. *ACS Catal.* **4,** 3644–3653 (2014).

4. He, Z. Q., Wang, D., Fang, H. Y., Chen, J. M. & Song, S. Highly efficient and stable Ag/$AgIO_3$ particles for photocatalytic reduction of CO_2 under visible light. *Nanoscale* **6,** 10540 (2014).

5. Wang, D., Yu, Y., Zhang, Z., Fang, H., Chen, J., He, Z. & Song, S. Ag/Ag_2SO_3 plasmonic catalysts with high activity and stability for CO_2 reduction with water vapor under visible light. *Environ. Sci. Pollut. Res.* **23,** 18369–18378 (2016).

6. Li, X., Zhuang, Z., Li, W. & Pan, H. Photocatalytic reduction of CO_2 over noble metal-loaded and nitrogen-doped mesoporous TiO_2. *Appl. Catal. A Gen.* **429–430,** 31–38 (2012).

7. Kong, D., Tan, J. Z. Y., Yang, F., Zeng, J. & Zhang, X. Electrodeposited Ag nanoparticles on TiO_2 nanorods for enhanced UV visible light photoreduction CO_2 to CH_4. *Appl. Surf. Sci.* **277,** 105–110 (2013).

8. Collado, L., Jana, P., Sierra, B., Coronado, J. M., Pizarro, P., Serrano, D. P. & de la Peña O'Shea, V. A. Enhancement of hydrocarbon production via artificial photosynthesis due to synergetic effect of Ag supported on TiO_2 and ZnO semiconductors. *Chem. Eng. J.* **224,** 128–135 (2013).

9. Chen, X., Wang, J., Huang, C., Zhang, S., Zhang, H., Li, Z. & Zou, Z. Barium zirconate: a new photocatalyst for converting CO_2 into hydrocarbons under UV radiation. *Catal. Sci. Technol.* **5,** 1758–1763 (2015).

10. Cheng, X., Dong, P., Huang, Z., Zhang, Y., Chen, Y., Nie, X. & Zhang, X. Green synthesis of plasmonic Ag nanoparticles anchored TiO_2 nanorod arrays using cold plasma for visible-light-driven photocatalytic reduction of CO_2. *J. CO_2 Util.* **20,** 200–207 (2017).

11. Li, K., Peng, T., Ying, Z., Song, S. & Zhang, J. Ag-loading on brookite TiO_2 quasi nanocubes with exposed {210} and {001} facets: Activity and selectivity of CO_2 photoreduction to CO/CH_4. *Appl. Catal. B Environ.* **180,** 130–138 (2016).

12. Bondarenko, A. S. & Ragoisha, G. A. Variable Mott-Schottky plots acquisition by potentiodynamic electrochemical impedance spectroscopy. *J. Solid State Electrochem.* **9,** 845–849 (2005).

13. Kennedy, J. H. Flatband Potentials and Donor Densities of Polycrystalline α-Fe_2O_3 Determined from Mott-Schottky Plots. *J. Electrochem. Soc.* **125,** 723 (1978).

14. Fujishima, A., Zhang, X. & Tryk, D. A. TiO_2 photocatalysis and related surface phenomena. *Surf. Sci. Rep.* **63,** 515–582 (2008).

15. Ioannides, T. & Verykios, X. E. Charge Transfer in Metal Catalysts Supported on Doped TiO_2: A Theoretical Approach Based on Metal–Semiconductor Contact Theory. *J. Catal.* **161,** 560–569 (1996).

16. Habisreutinger, S. N., Schmidt-Mende, L. & Stolarczyk, J. K. Photocatalytic reduction of CO_2 on TiO_2 and other semiconductors. *Angew. Chemie - Int. Ed.* **52,** 7372–7408 (2013).
